# Supplementary material for: Manx shearwater (Puffinus puffinus) rafting behaviour revealed by GPS tracking and behavioural observations
Source: PeerJ. 2019 Oct 21;7:e7863. doi: 10.7717/peerj.7863 (PMC6812691; doi:10.7717/peerj.7863)
Supplement: Equation S1 [file peerj-07-7863-s001.docx]

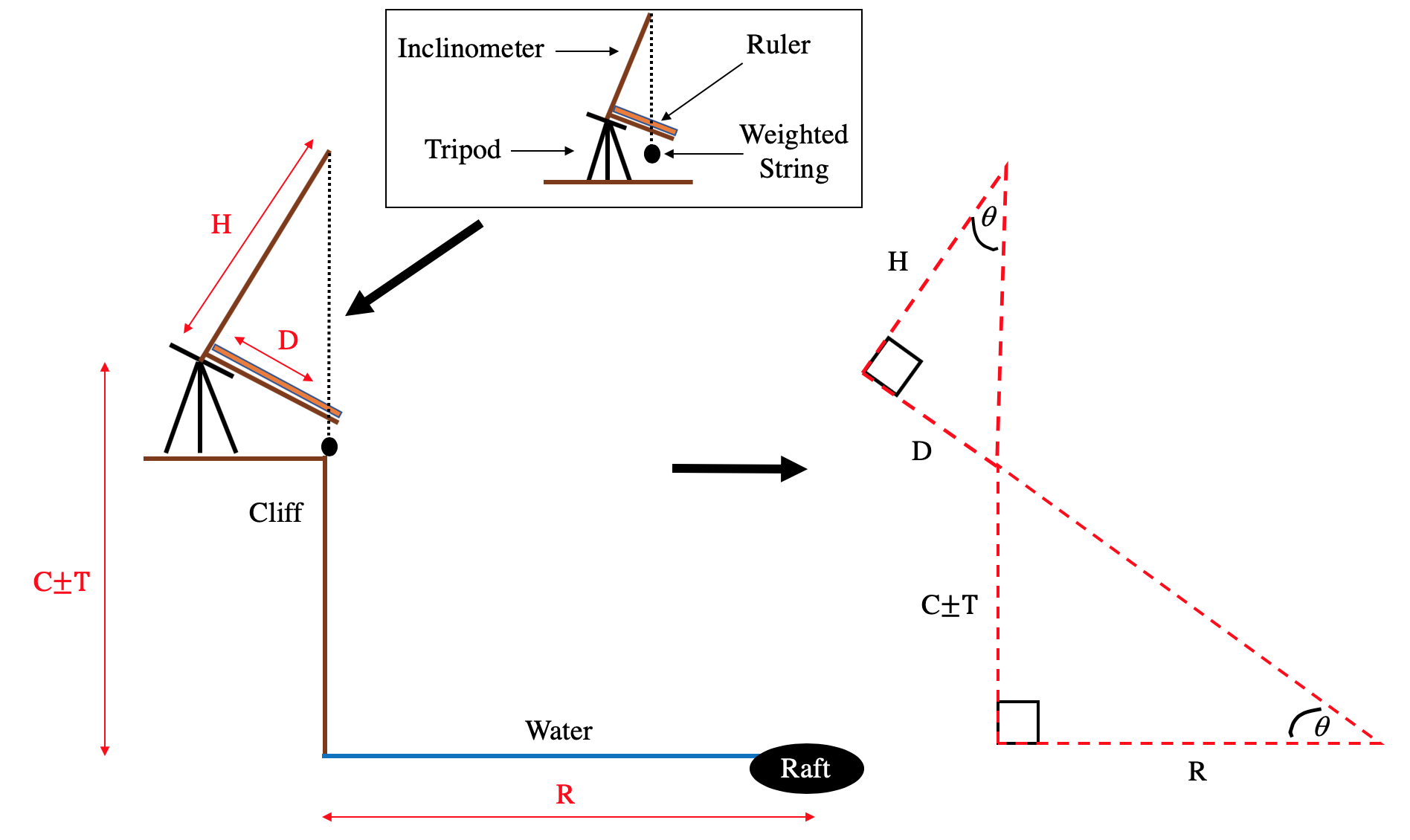


Illustration of equipment and method used to calculate Manx shearwater raft distance from shore. Where C = height of cliff at observation station plus tripod height (Garland Stone: 64.1 m; Skomer Head: 66.7 m; South Haven: 63.7 m; North Haven: 49.0 m), T = tide height relative to mean sea level, D = declination distance, H = height of inclinometer (0.895 m), and R is raft distance.

The inclinometer was mounted to a tripod which allowed it to be tilted and pointed towards a raft of Manx shearwaters. The weighted string then indicated a distance on the ruler which gave declination distance (D).

Because they are similar triangles and $\tan= \frac{\mathrm{Opposite}}{\mathrm{Adjacent}}$ :

$$Large Triangle: tan = \frac{(C\pm T)}{R}$$

$$Small Triangle: tan = \frac{D}{H}$$

$$tan = \frac{(C\pm T)}{R} = \frac{D}{H}$$

$$\frac{(C\pm T)}{R} \times R = \frac{D}{H} \times R$$

$$\left( C\pm T \right)\times H= \frac{D \times R}{H} \times H$$

$$\frac{\left( C\pm T \right) \times H}{D}= \frac{D \times R}{\text{D}}$$

$$\boldsymbol{Raft distance}\left( \boldsymbol{R} \right)\boldsymbol{=}\frac{\left( \boldsymbol{C\pm T} \right)}{\boldsymbol{D}}\boldsymbol{\times H}$$
